# The effects of cardiorespiratory fitness and acute aerobic exercise on executive functioning and EEG entropy in adolescents

**DOI:** 10.3389/fnhum.2015.00538

**Published:** 2015-10-19

**Authors:** Michael J. Hogan, Denis O’Hora, Markus Kiefer, Sabine Kubesch, Liam Kilmartin, Peter Collins, Julia Dimitrova

**Affiliations:** ^1^School of Psychology, NUIGalway, Ireland; ^2^Department of Psychiatry, University of UlmUlm, Germany; ^3^Transfer Center for Neuroscience and Learning, University of UlmUlm, Germany; ^4^Institute Education plusHeidelberg, Germany; ^5^College of Engineering and Informatics, NUIGalway, Ireland; ^6^Faculty of Medicine and Health Sciences, University of NottinghamNottingham, UK; ^7^Temerty Centre for Therapeutic Brain Intervention, Centre for Addiction and Mental HealthToronto, Canada

**Keywords:** EEG, entropy, exercise, cognition, fitness

## Abstract

The current study examined the effects of cardiorespiratory fitness, identified with a continuous graded cycle ergometry, and aerobic exercise on cognitive functioning and entropy of the electroencephalogram (EEG) in 30 adolescents between the ages of 13 and 14 years. Higher and lower fit participants performed an executive function task after a bout of acute exercise and after rest while watching a film. EEG entropy, using the sample entropy measure, was repeatedly measured during the 1500 ms post-stimulus interval to evaluate changes in entropy over time. Analysis of the behavioral data for lower and higher fit groups revealed an interaction between fitness levels and acute physical exercise. Notably, lower fit, but not higher fit, participants had higher error rates (ER) for No Go relative to Go trials in the rest condition, whereas in the acute exercise condition there were no differences in ER between groups; higher fit participants also had significantly faster reaction times in the exercise condition in comparison with the rest condition. Analysis of EEG data revealed that higher fit participants demonstrated lower entropy post-stimulus than lower fit participants in the left frontal hemisphere, possibly indicating increased efficiency of early stage stimulus processing and more efficient allocation of cognitive resources to the task demands. The results suggest that EEG entropy is sensitive to stimulus processing demands and varies as a function of physical fitness levels, but not acute exercise. Physical fitness, in turn, may enhance cognition in adolescence by facilitating higher functionality of the attentional system in the context of lower levels of frontal EEG entropy.

## Introduction

Research suggests that both acute bouts of exercise and higher levels of physical fitness may enhance cognitive functioning (Colcombe and Kramer, 2003; Stroth et al., 2009). Physical fitness is defined as the ability to carry out tasks without undue fatigue. The health-related components of physical fitness are cardiorespiratory fitness, muscular fitness, muscular strength, body composition, and flexibility. Agility, balance, coordination, speed, power, and reaction time are part of the skill-related physical fitness components (Caspersen et al., 1985). Cardiorespiratory fitness, the body’s ability to keep up with exercise that challenges the cardiorespiratory system (heart, lungs, blood vessels) for extended periods of time, is not only an important component of physical fitness but is also related to enhanced cognitive functioning (Hillman et al., 2008; McAuley et al., 2013). Building upon previous research in the area, the current study focused on the relationship between physical fitness, acute aerobic exercise and cognitive and brain functioning in adolescents using a measure of cardiorespiratory fitness, obtained with a maximal continuous graded exercise test performed on a cycle ergometer.

Developing and maintaining physical fitness, and cardiorespiratory fitness in particular, requires regular bouts of acute exercise. Similar to the effects of physical fitness, acute bouts of exercise have been associated with enhanced cognitive functioning. Notably, results from a recent meta-analysis highlighted that the cognitive benefits of 20 min of acute exercise are largest for school age children (including adolescents) relative to the population as a whole (Chang et al., 2012). However, less is known about the electrophysiological mechanisms associated with the positive effects of exercise on cognitive performance in adolescents, and whether or not the effects of acute exercise are any different for higher fit and lower fit adolescents. The current study uses electroencephalography (EEG), that is, the recording of electrical activity along the scalp, to examine if cardiorespiratory fitness levels or acute bouts of aerobic exercise influence the entropy of the EEG in response to cognitive demands in adolescents, or whether fitness levels interact with acute exercise to influence EEG entropy and cognitive performance in this group.

Notably, both correlational and experimental studies have demonstrated global cognitive benefits of physical exercise in children and adolescents (Sibley and Etnier, 2003). Exercise programmes across several weeks have also been shown to improve cognitive functioning in children and adolescents (Tuckman and Hinkle, 1986; Hinkle et al., 1993; Davis et al., 2007). A number of studies in this area have focused on executive functioning, defined as the ability to coordinate cognition and action (Norman and Shallice, 1986) and support action planning, switching, and inhibition (Royall et al., 2002). In a study that sought to identify electrophysiological mechanisms associated with the positive effects of exercise on cognitive performance in children, Hillman et al. (2009) examined executive functioning and event-related potential (ERP) differences between higher- and lower-fit pre-adolescent children (average age 9.4 years). They found that higher fitness levels were associated with superior executive functioning performance as measured using the Erikson flanker task, and also with larger P3 ERP amplitudes in response to stimuli. Hillman and colleagues suggested that increased allocation of attentional resources, as indicated by larger P3 amplitudes during the encoding of stimuli, was related to better performance in the more physically fit children (see also Hillman et al., 2005). Research studies confirm that other ERP components associated with executive control including the N2 ERP component are modulated by higher levels of physical fitness in adolescence (Themanson et al., 2006; Stroth et al., 2009). In addition to fitness levels, acute exercise manipulations have been shown to affect oscillatory activity in the human EEG (Moraes et al., 2007; Bailey et al., 2008). For example, studies have reported that exercise increases oscillatory activity in the alpha range during subsequent cognitive performance (Petruzzello and Landers, 1994). However, very little is known about the effects of fitness levels and acute exercise on other EEG measures, such as EEG entropy.

Historically, entropy is one of the most well established metrics for quantifying the uncertainty of any (bio-)signal. First proposed by Boltzmann [1844–1906], entropy is a measure of the number of microscopic ways that a certain macroscopic state can be realized. This concept was further extended by Shannon (Shannon and Weaver, 1963) to the information theory domain in proposing that the information gained when a measurement is taken depends on the number of possible outcomes, of which only one is realized. Systems whose underlying dynamics are more unpredictable will have greater entropy, whereas lower entropy systems by definition are more predictable. As such, EEG entropy measures can be used to provide an index of complexity that is equivalent to measuring the uncertainty or lack of regularity in a signal (Rezek and Roberts, 1998; Bhattacharya, 2000) and can provide a window into levels of adaptive and maladaptive system uncertainty at a brain level that are predictive of key performance differences (Rosso et al., 2011).

Entropy metrics are potentially useful markers of the enhancement of cognitive functioning due to both acute bouts of exercise and higher levels of physical fitness. For example, theoretical models of cognitive performance suggest that an increase in the level of intra-network variability may be causally related to poorer cognitive performance (Li and Lindenberger, 1998; Li et al., 2006). Notably, Li and colleagues demonstrated that there was a greater disruption to a subject’s engaged performance as the signal-to-noise ratio (SNR) of the system decreased. One potential implication of this observed reduction in the SNR is a generalized reduction in adaptive system uncertainty (i.e., the range of possible states that the system can achieve in response to adaptive demands is reduced) in the context of executive functioning tasks (i.e., less overall capacity to coordinate cognition and action (Norman and Shallice, 1986) and support action planning, switching, and inhibition (Royall et al., 2002)). One possibility is that acute exercise and fitness influence adaptive system uncertainty, which in turn influences executive functioning.

Importantly, recent research suggests that EEG entropy may be sensitive to cognitive demands and can be used to predict group differences in cognitive performance (Hogan et al., 2012; O’Hora et al., 2013). For example, O’Hora et al. (2013) found that task sensitivity of frontal EEG during encoding predicted later retrieval in a sample of younger and older adults, with reduced task sensitivity of frontal EEG observed in older adults with cognitive decline. However, no study to date has examined if fitness levels or acute bouts of aerobic exercise influence the entropy of the EEG in response to cognitive demands, or whether fitness levels interact with acute exercise in this context. It has been suggested that physical exercise may improve cognition by modulating temporal functional dynamics and connections between cell assemblies that support task performance (Hogan et al., 2013). Although the operation of neural assemblies is difficult to capture in real time, the millisecond temporal resolution of EEG and the use of entropy analysis to examine the complexity of electrical activity across scalp locations, suggests that EEG entropy measures may offer insight into the operation of neural assemblies. Therefore, the current study aimed to establish whether cardiorespiratory fitness affects baseline event-related sample entropy during the performance of an executive functioning task and whether different patterns of event-related sample entropy change due to acute exercise would be observed in participants with different fitness levels.

In examining the effects of fitness and exercise on cognitive and neural function in the current study, we were sensitive to the fact that different brain regions have been implicated in different cognitive functions, for example, with frontal lobe involvement being identified as critical for performance on executive functioning tasks (West, 1996; Cabeza, 2002; Hogan et al., 2011). Therefore, to enhance our ability to estimate the effects of fitness and exercise, we measured average sample entropy (that is the sample entropy averaged across a group of regional electrode sites) across the frontal, temporal, and parietal lobes for both hemispheres, separately. In light of previous research on the role of the frontal lobe in executive functioning tasks (West, 1996; Cabeza, 2002), we explored the possibility that any differences apparent in the resultant sample entropy measures between higher fit and lower fit adolescents and between acute exercise and rest conditions would be largest in the frontal lobes. If such a regionally averaged sample entropy metric is an index of information complexity, and if both higher fitness levels and acute bouts of exercise increase information processing complexity, we predicted increases in the sample entropy measure from rest to acute exercise, higher entropy in higher fit relative to lower fit participants, and further increases in entropy in higher fit relative to lower fit participants in response to exercise. The alternative hypotheses with regards to EEG entropy was that greater effort would be required by the lower fit group and would therefore result in higher levels of information processing complexity and EEG entropy in response to the executive performance task used in the current study (i.e., the Erikson flanker task). Similarly, the alternative hypothesis predicted that the acute exercise condition would increase cortical processing efficiency and thus result in lower levels of EEG entropy in comparison with the rest condition.

In relation to behavioral measures, as physical fitness produced more consistent and robust effects in comparison to acute exercise in previous studies (Lardon and Polich, 1996; Themanson and Hillman, 2006), we expected that physical fitness would enhance behavioral performance to a greater extent than acute exercise. Specifically, it was hypothesized that higher physical fitness would be associated with shorter reactions times (RTs) and lower error rates (ER) compared to lower physical fitness. It was also hypothesized that acute aerobic exercise would positively affect performance to a lesser extent.

## Materials and Methods

### Participants

Thirty healthy adolescents participated in the present study. Participants were recruited through the local administration of secondary schools and were invited to participate in the study by means of an information event at school during class. Mean age was 14.2 years (SD = 0.5, range = 13–14 years). All participants were right-handed and had normal, or corrected to normal vision. Participants were carefully screened and did not show any signs of a history of neurological or psychiatric disorders or medication intake. Participants were divided into two groups according to a median split of the fitness distribution. This was performed for boys and girls separately in order to keep the gender distribution equal in each group. Fifteen adolescents (ten boys and five girls) were classified as “higher fit” and fifteen adolescents (nine boys and six girls) were classified as “lower fit”. With regard to participants’ age, height, and weight no significant differences between the groups existed. All adolescents received information material in order to fully inform their parents. They were allowed to participate after their legal guardian had permitted informed consent. The present study was carried out in accordance with the ethical review board at the University of Ulm, Germany.

### Study Design

During a regularly scheduled physical education class, participants underwent a maximal incremental cycling test on an electrically braked stationary cycle ergometer to assess physical fitness via individual maximal exercise performance. This exercise test was conducted in order to plan an individually adjusted bout of exercise with heart rate control. Bouts of exercise were planned at 60% of the individual’s maximal heart rate, representing about 50–60% of maximum oxygen uptake (Wasserman and McIlroy, 1964), leading to a workout at a moderate but brisk intensity.

**Figure 1:**
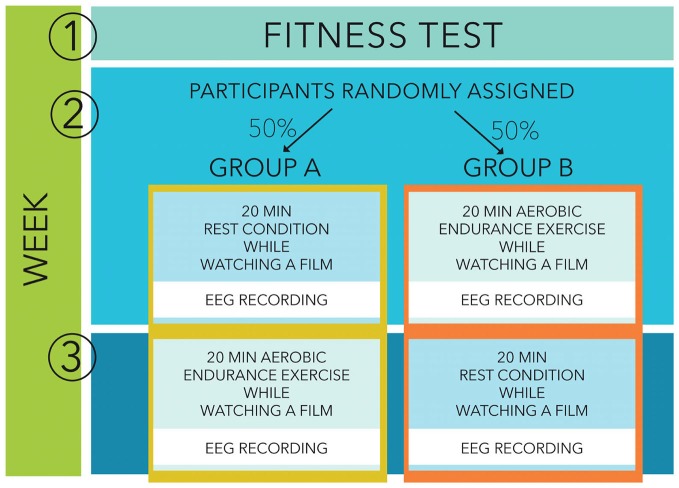
**Study design**.

Participants were then randomly assigned to the study with two separate recording sessions, one following a 20 min bout of moderate aerobic exercise and one following a 20 min period of rest (see Figure 1). One group started with an exercise condition (week 2) followed by a rest condition (week 3) while the other group started with a rest condition followed by an exercise session. During both sessions, participants came into the laboratory and watched a movie, during the cycling workout as well as during the resting condition. They participated in both conditions in a random order within an exact 7 days interval, at the same day of the week, at the same time of the day to avoid differences in preceding activities or circadian distortions. During both sessions, participants were prepared for the EEG recordings and subsequently performed the 20 min exercise condition or the resting condition, sitting on the cycling ergometer for 20 min in both conditions to keep them as similar as possible. Afterwards they performed an Eriksen flanker task with EEG recordings.

### Fitness Testing

To estimate cardiorespiratory fitness, a component of physical fitness, we used a maximal continuous graded exercise test performed on a cycle ergometer until voluntary exhaustion. With such a protocol, there is a high correlation between exercise time and directly measured maximal oxygen uptake, allowing for an estimation of fitness (Gupta et al., 2011). The fitness test was performed 1 week before the recording sessions started. The participating adolescents completed a continuous graded maximal exercise test during a regular school day physical education class, with the test administered by members of the research team. The testing protocol started with a resistance of 25 W. After every two minutes, the watt-load of the dynamometer was then increased by 25 W, while the participant maintained the pedalling rate constant at 60 rotations per minute. Grades were continuously increased until the participant reached subjective exhaustion and stopped pedalling. At each interval (every two minutes) the investigator recorded heart rates from the monitor the subject was wearing (Polar Electro^®^, Buettelborn, Germany, Model F6). Heart rate at each interval up to maximal heart rate, absolute time pedalling on the bike (in seconds) as well as maximal watt performance was documented on a record sheet for each subject separately. Maximal watt performance on the dynamometer was then related to the body mass index (BMI) to establish a standardized value for physical fitness (fitness = Watt performance/BMI), controlling for body mass and size (Armstrong and Welsman, 2007). Participants were then divided by means of a median split into relatively higher fit and lower fit groups within our sample. However, as fitness norms are not available for this test, the absolute fitness level cannot be determined in our participants. The median fitness score for girls was 7.11 and the median fitness score for boys was 8.42. For participants’ demographic variables and fitness parameters see Table 1.

**Table 1:** **Means (SDs) of participants’ demographic and exercise variables for higher fit and lower fit groups separately, and *p*-value for *t*-test differences between groups**.

	Higher fit (*N* = 15)	Lower fit (*N* = 15)	*p*-value
Age (years)	14.29 (0.48)	14.32 (0.70)	0.86
Bodyweight (kg)	50.81 (9.41)	56.63 (16.25)	0.24
Height (cm)	1.64 (0.07)	1.61 (0.09)	0.45
BMI	19.49 (3.44)	19.90 (3.18)	0.73
Max. duration of exercise (seconds)	616 (154.5)	546 (157.8)	0.22
Max. watt performance of exercise	168 (31.99)	138 (24.76)	0.008
Watt/BMI ratio	8.91 (1.21)	6.78 (0.87)	<0.0001

### Cognitive Task

We combined a Go/No Go task with an Eriksen flanker paradigm, which has been used in several earlier studies (e.g., Ruchsow et al., 2005). Eight different letter strings (congruent: BBBBB, DDDDD, VVVVV, and UUUUU; incongruent: BBDBB, DDBDD, UUVUU, and VVUVV) were presented on a computer screen in randomized order. Subjects had to focus on the target letter in the middle of an array and had to press a response key upon the appearance of the letters B and U (Go-condition) and to withhold key press upon the appearance of D and V (No Go-condition). Instructions equally emphasized speed and accuracy. Responses were executed with the index finger of their dominant hand. Letter strings were preceeded by a warning stimulus (fixation cross) presented for 600 ms (ms) centrally on the screen before the target stimuli appeared. Each letter combination was presented for a total of 480 ms, with the target letter in the middle of the array appearing after a 320 ms delay with a duration of 80 ms. Subjects received feedback according to their performance 750 ms after key press. As feedback stimuli we used the German expressions for “correct”, “false”, and “faster”. Feedback stimuli were presented for 500 ms. The inter-trial-interval was 2600 ms. In each trial, participants received a reward for correct responses, and were penalized for errors (5 points per trial). In the end, the greatest amount of points was calculated and the winner was promised a reward. Before the main experiment, subjects had a training period of 12–20 trials. The whole experiment consisted of five blocks of 120 trials each (300 Go-trials; 300 No Go-trials). The behavioral data collected was response latency in ms from the presentation of the target stimulus (Go-trials) and response accuracy in terms of percentage of correct responses (Go- and No Go-trials). Participants were seated in a comfortable chair in a sound-attenuating, electrically shielded booth. The whole experiment lasted about 3 h, including exercise/rest sessions, pauses, electrode placement, and removal of electrodes.

### EEG Recording and Entropy Calculation

EEG was continuously recorded using 39 channels mounted in an elastic cap (Easy Cap, Herrsching, Germany). Electrodes were positioned according to the extended 10–20 system. All electrodes were referenced to an electrode at the left earlobe and re-referenced to average reference off-line. Eye movements were registered by vertical and horizontal EOG. Electrode impedances were kept below 5 kΩ. The EEG was amplified by Neuroscan Synamps amplifiers (bandwidth DC-50 Hz; 50 Hz notch filter) and A/D converted with 12-bit resolution at a rate of 250 Hz. Subsequent standard pre-processing of the EEG was carried out to remove ocular and other artifacts from the raw EEG samples. This process resulted in data for some subject trials being discarded as they were too contaminated with artifacts for the artifact removal algorithms to be successfully applied. The remaining EEG data was then epoched using the stimulus presentation point as reference.

Incorrect responses were excluded from the analysis. The data of participants with fewer than 10 usable epochs for each condition type were removed from the analysis. This led to the removal of three participants. The remaining epochs were separated into stimulus categories. The entropy analysis was completed by repeatedly applying a 500 ms duration rectangular window to the samples in each epoch on each recorded EEG channel. The center point of this window was initially located at +250 ms post-stimulus and then moved in steps of 100 ms; the final position of this window would encompass the +1000 ms to +1500 ms post-stimulus period. It should be noted that any reference hereafter to the temporal location of the window of samples for which the sample entropy was calculated refers to the location of the center of the window of samples which were analyzed.

The sample entropy was calculated for each of the above windows of samples (for each channel in each epoch). Entropy measures can be used to quantify the uncertainty of any (bio-) signal. Shannon defined entropy in terms of the probabilities of the system being in each of the allowed system “micro-states” (s_i_), as in (1).

(1) $$H(s)\;=\;\sum_{i\;=\;1}^{N}-p(s_{i})\ln p(s_{i})$$

A significant practical challenge in the application of this definition of entropy to EEG waveforms relates to the estimation of the probabilities of the micro-states of a system, *p*(s_i_). A variety of approaches to address this problem have been reported in the literature; these include approaches based on the implementation of time-frequency based decompositions of the EEG in order to construct a pseudo-probability distribution function (Rosso et al., 2002) and the estimation of a probability distribution function using histogram calculated from the time domain EEG samples (Lofgren et al., 2007; Zhao et al., 2007). Richman and Moorman (2000) proposed an alternative time domain based approach which avoided this issue and which has been successfully applied to different bio-signals, namely sample entropy. This method of quantifying a signal’s entropy is based on identifying and then quantifying repetitions of similar sequences in a signal. In terms of an EEG signal, this formulation of entropy estimates the uncertainty within an EEG signal epoch consisting of *N* samples. The sample entropy (sampEn) of such an epoch of EEG samples is calculated as the log likelihood that a block of m consecutive EEG sample values (from the epoch of *N* samples) which are *similar* (i.e., within a tolerance value r using a defined distance measure e.g., Euclidean distance) remain *similar* when the block size is increased to *m* + 1 samples, as in (2).

(2) $$sampEn(m,r,N)\;=\;-\ln\left(\frac{U_{m+1}}{U_{m}}\right)$$

where U_m_ is the conditional probability that the block of *N* samples is similar for a match length of m samples. Abásolo et al. (2006) applied this estimator to EEG entropy analysis and it is this measure that is utilized in this study.

In the current study, a number of different combinations of the (*r*, *m*) values used within the sample entropy calculation were evaluated. The results reported here represent those obtained for (*r* = 0.25, *m* = 2), where the value of “*r*” refers to a multiplier value for the standard deviation of the sample values in the frame being analyzed. Results obtained for other combinations of (*r*, *m*) values showed little significant deviation from the results reported in this work. The next stage of processing of the sample entropy values for a given EEG channel was to determine (for each subject) an average sample entropy value (calculated for each window location) across all epochs of a given stimulus type. Using these values, the final stage of processing involved the calculation of the mean sample entropy value across six regional electrode groups. The electrode groups which were used in this stage covered the left frontal (Fp1, F3, F7), right frontal (Fp2, F4, F8), left temporal (T7, TP7, FT7), right temporal (T8, TP8, FT8), left parietal (P3, P7) and right parietal (P4, P8) regions.

## Results

### Behavioral Performance

It was expected that greater physical fitness and acute physical exercise would enhance cognitive performance. Two measures of cognitive performance during the flanker task were employed: reaction time and errors. For both dependent variables, lower values indicated better performance. Behavioral measures (reaction time, error rate) were analyzed using two 2 (Fitness: low vs. high) × 2 (Exercise: rest vs. exercise) × 2 (Trialtype: Go vs. No Go) × 2 (Congruency: congruent vs. incongruent) ANOVAs.

**Figure 2:**
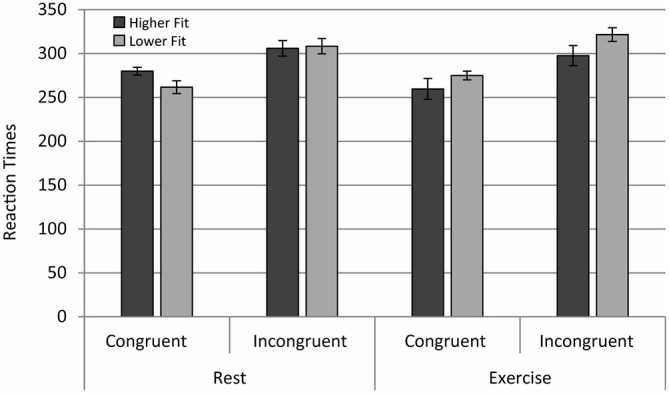
**Reaction times for the higher fit and lower fit groups for the rest and exercise conditions for both congruent and incongruent trials**.

A repeated measures ANOVA on the mean RT (reaction time) of hits (correct Go-responses) revealed a main effect of congruency, *F*_(1,28)_ = 53.60, *p* < 0.001. In congruent trials, RT was significantly faster than in incongruent trials, the classic “flanker effect”. In contrast to our expectations, no main effects of fitness level or acute exercise on RT were observed. A significant Fitness × Exercise effect was observed, *F*_(1,28)_ = 7.27, *p* = 0.012. Low Fitness participants exhibited longer RTs in the acute exercise condition compared to the rest condition, but this effect was not statistically significant, *F*_(1,28)_ = 3. 37, *p* = 0.07. In contrast, higher fit participants had significantly faster RTs in the exercise condition in comparison with the rest condition, *F*_(1,28)_ = 3.90, *p* = 0.05; see Figure 2. In summary, the effects of acute exercise depended upon fitness, enhancing the performance of fitter participants and degrading the performance of less fit participants.

**Figure 3:**
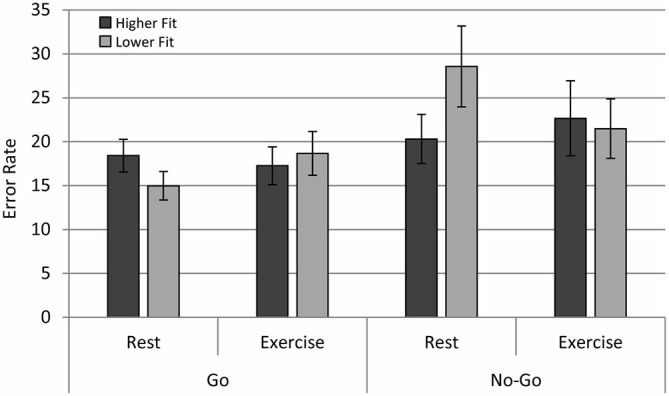
**Error rates (ER) for the higher fit and lower fit groups for the rest and exercise conditions for both Go and No Go trials**.

Analysis of ER across conditions revealed a main effect of congruency, *F*_(1,28)_ = 51.40, *p* < 0.0001, with lower ER observed in congruent (*M* = 7.20, SD = 3.84) relative to incongruent trials (*M* = 13.10, SD = 7.48), a flanker effect on accuracy. There was a main effect of Trialtype, *F*_(1,28)_ = 5.037, *p* < 0.05, with lower ER for Go trials relative to No Go trials. In contrast with expectations, there were no main effects of physical fitness and physical exercise on accuracy. The interactions Exercise × Trialtype, *F*_(1,28)_ = 4.54, *p* < 0.05; and Fitness × Exercise × Trialtype, *F*_(1,28)_ = 17.31, *p* < 0.001 were significant. The results of follow-up ANOVAs conducted for Higher Fitness and Lower Fitness groups separately revealed a significant Exercise × Trialtype effect for the Lower Fitness group, *F*_(1,14)_ = 16.90, *p* < 0.001, and a non-significant Exercise × Trialtype effect for the Higher Fitness group, *F*_(1,14)_ = 2.430, *p* = 0.141. *Post hoc* contrasts revealed that participants in the Lower Fitness group showed significantly higher ER for No Go relative to Go trials in the rest condition, *F*_(1,28)_ = 11.68, *p* < 0.005; see Figure 3. In summary, physical fitness and exercise affected aspects of cognitive performance, but the effects were more complex than expected.

### EEG Entropy

**Figure 4:**
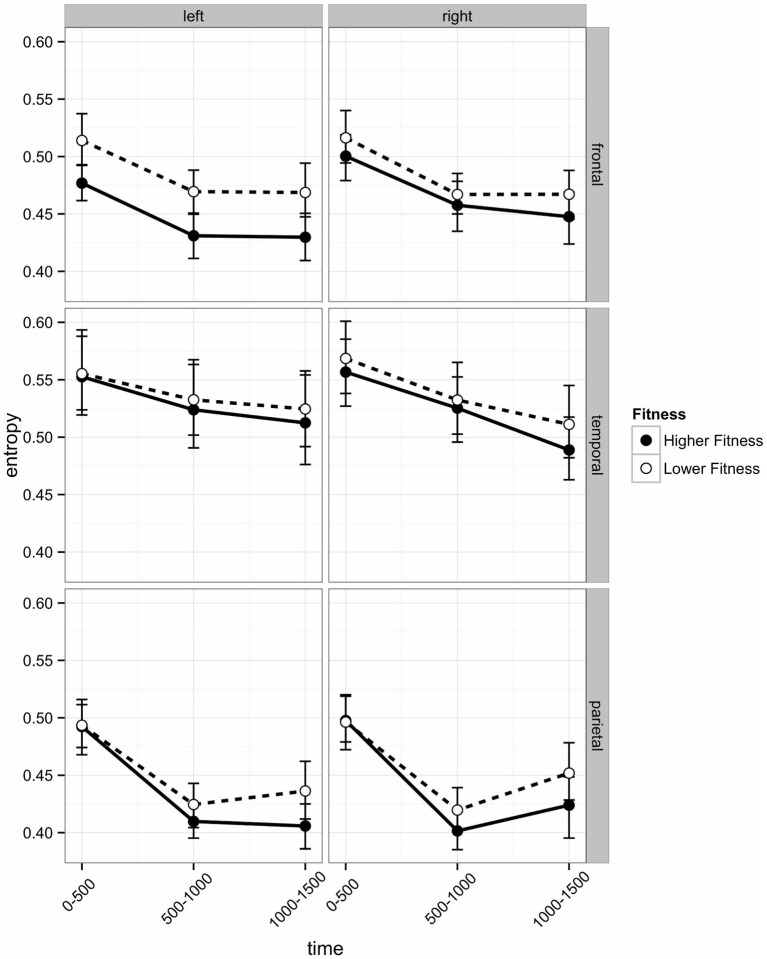
**Post-stimulus EEG entropy for left and right hemispheres across three regions of interest (frontal, temporal, and parietal) of participants in the higher fit and lower fit groups.** Labels on the *x* axis denote 500 ms time intervals post-stimulus (see text for details). Error bars are bootstrapped confidence intervals. Plots were developed using Hmisc (Harrell, 2014) and ggplot2 (Wickham, 2009) packages in R Core Team (2014).

EEG entropy was calculated across three regions of interest (ROI) frontal, temporal, and parietal—in both left and right hemispheres. Entropy was binned in 500 ms periods (0–500 ms, 500–1000 ms, 1000–1500 ms), which roughly correspond to the following sequence of events in one experimental trial: The 0–500 ms bin covers the stimulus processing, decision and response execution period, the 500–1000 ms bin includes feedback expectation, and the 1000–1500 ms bin covers processing of the feedback itself. The length of the bins ensures sufficient consecutive data points (125; sampling rate 250 Hz). Initial analyses indicated that the post-stimulus change in entropy across these three time intervals varied across ROIs. As can be seen in Figure 4, there was a linear decrease across intervals in the temporal region, but frontal and parietal entropy decreased sharply from the post-stimulus interval to the 500–1000 ms interval. Entropy remained at this lower level in the frontal region, but in the parietal region, entropy rebounded from the second interval (500–1000 ms) to the third (1000–1500 ms), particularly in the right hemisphere.

Linear mixed effects models (lme4; Bates et al., 2014) were employed to model the change in entropy across time intervals. This analysis strategy takes advantage of the multiple data points available per participant and facilitated modeling different patterns of time-related change. Due to the different effects of time across ROIs, separate analyses of each ROI were conducted. For ease of comparison, a consistent model structure was employed across regions; the models included random intercepts and random linear and quadratic estimates of Time for each participant and fixed effects of linear Time, quadratic Time, Hemisphere, Fitness, Exercise, Trialtype, Congruency and all interactions. To test for significant effects these fixed effects and their associated interactions were gradually removed from the model (see Tables 2 and 3). All *p* values were adjusted according to Hochberg (1988).

**Table 2:** **Main and interaction effects of factors retained in the Frontal and Temporal region models**.

	Frontal				Temporal			
	*b*	*SE*	*t*	*p*	*b*	*SE*	*t*	*p*
(Intercept)	0.4562	0.0065	69.7449		0.5285	0.0112	47.0183	
Hemisphere	**−0.0120**	**0.0020**	**−6.0528**	**0.004**	−0.0006	0.0028	−0.2219	0.8974
Time	**−0.0198**	**0.0012**	**−17.2521**	**0.004**	**−0.0200**	**0.0012**	**−17.2913**	**0.005**
Time Sq	**0.0142**	**0.0022**	**6.3401**	**0.004**	0.0035	0.0013	2.6770	0.0666
Fitness	−0.0239	0.0131	−1.8241	0.4428	−0.0079	0.0225	−0.3511	0.8974
Hemisphere × Time	0.0019	0.0011	1.6906	0.4545	**0.0111**	**0.0016**	**6.8850**	**0.005**
Hemisphere × Time Sq	0.0010	0.0016	0.6176	0.7507	0.0037	0.0023	1.6277	0.6216
Hemisphere × Fitness	**−0.0290**	**0.0040**	**−7.2959**	**0.004**	−0.0016	0.0056	−0.2857	0.8974
Time × Fitness	−0.0011	0.0023	−0.4756	0.7507	−0.0040	0.0023	−1.7445	0.6216
Time Sq × Fitness	−0.0027	0.0045	−0.5992	0.7507	−0.0029	0.0026	−1.1000	0.8974
Hemisphere × Time × Fitness	0.0007	0.0023	0.3177	0.7507	0.0004	0.0032	0.1290	0.8974
Hemisphere × Time Sq × Fitness	0.0058	0.0033	1.7879	0.4428	0.0075	0.0046	1.6503	0.6216

*Numbers in bold denote significant effects*.

**Table 3:** **Main and interaction effects of factors retained in the Parietal region model**.

	Parietal			
	*b*	*SE*	*t*	*p*
(Intercept)	0.4139	0.0061	67.9005	
**Hemisphere**	**0.0066**	**0.0018**	**3.7278**	**0.0046**
**Time**	**−0.0267**	**0.0023**	**−11.5451**	**0.009**
**Time Sq**	**0.0322**	**0.0025**	**12.6503**	**0.009**
Fitness	−0.0164	0.0122	−1.3481	0.986
Go-No Go	−0.0033	0.0018	−1.8945	0.9312
**Hemisphere × Time**	**−0.0052**	**0.0010**	**−5.1444**	**0.009**
**Hemisphere by Time Sq**	**−0.0113**	**0.0014**	**−7.8936**	**0.009**
Hemisphere × Fitness	0.0034	0.0035	0.9715	0.986
Time × Fitness	−0.0120	0.0046	−2.5883	0.1632
Time Sq × Fitness	0.0013	0.0051	0.2459	0.986
Hemisphere × Go-No Go	−0.0026	0.0035	−0.7380	0.986
**Time × Go-No Go**	**−0.0036**	**0.0010**	**−3.5873**	**0.0066**
Time Sq × Go-No Go	−0.0013	0.0014	−0.9275	0.986
Fitness × Go-No Go	0.0022	0.0035	0.6328	0.986
Hemisphere × Time × Fitness	−0.0000	0.0020	−0.0176	0.986
Hemisphere × Time Sq × Fitness	−0.0040	0.0029	−1.3794	0.986
Hemisphere × Time × Go-No Go	−0.0029	0.0020	−1.4197	0.986
Hemisphere × Time Sq × Go-No Go	−0.0015	0.0029	−0.5208	0.986
Hemisphere × Fitness × Go-No Go	−0.0003	0.0070	−0.0455	0.986
Time × Fitness × Go-No Go	−0.0032	0.0020	−1.5684	0.986
Time Sq × Fitness × Go-No Go	−0.0020	0.0029	−0.6996	0.986
Hemisphere × Time × Fitness by Go-No Go	0.0009	0.0041	0.2279	0.986
Hemisphere × Time Sq × Fitness by Go-No Go	0.0015	0.0057	0.2647	0.986

*Numbers in bold denote significant effects*.

In light of previous findings, we were particularly interested in whether EEG entropy would be increased following acute exercise. Exposure to acute exercise did not significantly affect EEG entropy in any of three ROIs in the current study. In the Frontal and Temporal ROIs, there were also no significant effects of Congruency or Trialtype. In the Parietal ROI, there were no significant effects of Congruency, but there were differences across Go and No Go trials (Trialtype) and this fixed effect was retained. The flanker effect was not observed in EEG entropy.

In the frontal region, there was a main effect of Hemisphere (*b* = −0.0120), with entropy on the left side significantly lower than on the right. There was a Hemisphere × Fitness interaction effect (*b* = −0.0290), with left frontal entropy significantly lower for higher fit participants relative to lower fit participants across all three time intervals (see Figure 4; 0–500 ms: *Z* = −2.32, *p* = 0.027; 500–1000 ms: *Z* = −2.32, *p* = 0.027; 1000–1500 ms: *Z* = −2.21*, p* = 0.027). There were no significant differences between higher fit and lower fit participants in the right hemisphere (*p* > 0.05 for all three interval comparisons).

Frontal EEG entropy changed across time, with significant linear (*b* = −0.0198) and quadratic (*b* = −0.0120) main effects of time observed. As can be seen in Figure 4, for both higher fit and lower fit participants, left and right frontal entropy decreased from the post-stimulus interval (0–500 ms) to the 500–1000 ms interval (Left: *M*_diff_ = −0.045, *t*_29_ = −11.24, *p* < 0.0005; Right: *M*_diff_ = −0.046, *t*_29_ = −12.86, *p* < 0.0005), but there was no difference between entropy in the 500–1000 ms interval and entropy in the 1000–1500 ms interval (Left: *M*_diff_ = −0.001, *t*_29_ = −0.24, *p* = 0.81; Right: *M*_diff_ = −0.004, *t*_29_ = −1.12, *p* = 0.55). A complete summary of the remaining non-significant effects can be found in Table 2.

Temporal EEG entropy decreased more gradually in the left temporal region than in the right temporal region (*b* = 0.0111) for both higher fit and lower fit participants (see Figure 4). In both hemispheres, entropy decreased from the post-stimulus interval (0–500 ms) to the 500–1000 ms interval (Left: *M*_diff_ = 0.026, *t*_29_ = 9.36, *p* < 0.0005; Right: *M*_diff_ = 0.034, *t*_29_ = 12.27, *p* < 0.0005), and from the 500–1000 ms interval to the 1000–1500 ms interval (Left: *M*_diff_ = 0.009, *t*_29_ = 3.22, *p* = 0.003; Right: *M*_diff_ = 0.028, *t*_29_ = 7.60, *p* < 0.0005).

**Figure 5:**
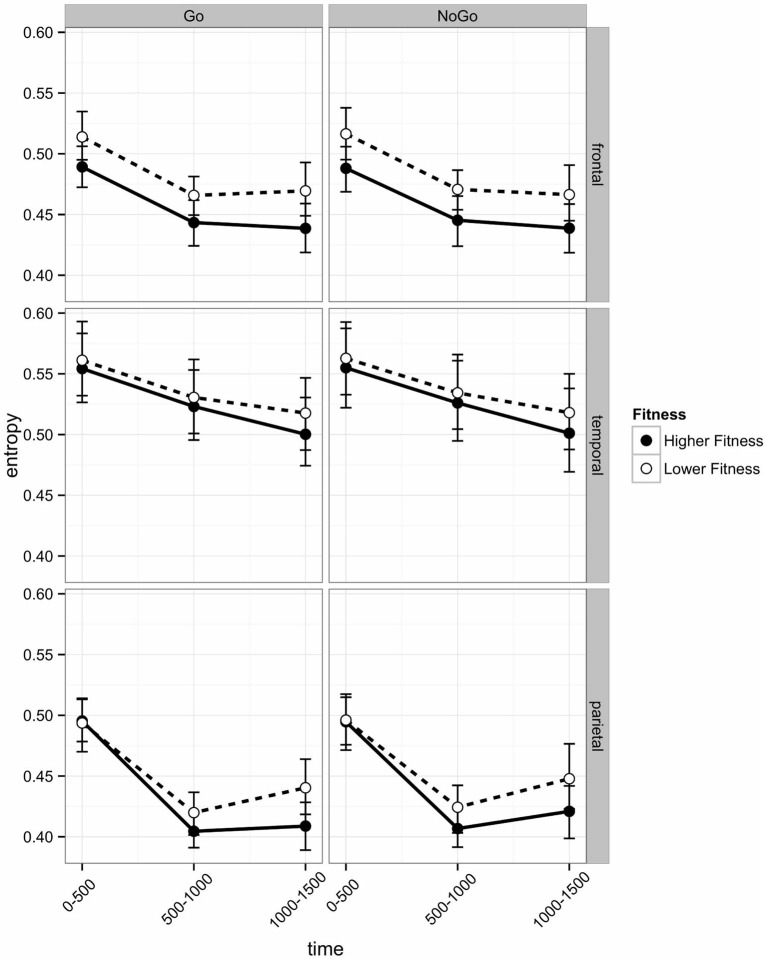
**Post-stimulus EEG entropy during Go and No Go trials across three regions of interest (frontal, temporal, and parietal) of participants in the higher fit and lower fit groups.** Labels on the *x* axis denote 500 ms time intervals post-stimulus (see text for details) and error bars indicate bootstrapped confidence intervals.

As can be seen in Figure 5 in the Parietal region, the change in entropy over Time was different across Go and No Go trials (*b* = −0.0036) and across Hemispheres (Hemisphere × Time: *b* = −0.0052; Hemisphere × Time Sq; *b* = −0.0113). *Post hoc* *t*-tests revealed differences in the time-related change in entropy across Go and No Go trials. In both Go and No Go trials, entropy reduced significantly from the post-stimulus interval to the 500–1000 ms interval (Go: *M*_diff_ = −0.081, *t*_29_ = −18.52, *p* < 0.0005; No Go: *M*_diff_ = 0.079, *t*_29_ = 18.18, *p* < 0.0005). The increase from the 500–1000 ms interval to the 1000–1500 ms interval was only significant for No Go stimuli (Go: *M*_diff_ = 0.013, *t*_29_ = 2.20, *p* = 0.11; No Go: *M*_diff_ = 0.019, *t*_29_ = 3.53, *p* = 0.007).

Differences in time-related change in entropy were observed across Hemispheres in the parietal region. Entropy reduced significantly from the post-stimulus interval to the 500–1000 ms interval in both hemispheres (Left: *M_diff_* = −0.075, *t*_29_ = −17.62, *p* = 0.003; Right: *M*_diff_ = −0.086, *t*_29_ = −16.49, *p* < 0.0005), but entropy increased significantly from the 500–1000 ms interval to the 1000–1500 ms interval on the right side (*M*_diff_ = 0.027, *t*_29_ = 4.03, *p* = 0.002), and not on the left (*M*_diff_ = 0.004, *t*_29_ = 0.82, *p* = 0.41). Main effects of linear (*b* = −0.0267) and quadratic time (*b* = 0.0322) were also observed. Entropy reduced from the post-stimulus interval to the 500–1000 ms interval then stabilized or rebounded from the 500–1000 ms interval to the 1000–1500 ms interval. A summary of effects can be found in Table 3.

## Discussion

The current study examined the EEG entropy and behavioral performance of higher fit and lower fit adolescent participants during an executive control task after both acute exercise and a resting condition watching a film. Analysis of behavioral data revealed an interaction between fitness levels and acute physical exercise for both RT and error rate data. Notably, lower fit, but not higher fit, participants had higher ER for No Go relative to Go trials in the resting condition. Furthermore, higher fit participants had significantly faster RTs in the exercise condition in comparison with the rest condition. Although there was a general flanker congruency effect in the RT data, this effect was neither modulated by fitness level nor by exercise.

Significant EEG entropy differences between higher fit and lower fit participants were also observed. Notably, in the frontal region, left hemisphere entropy was significantly lower for higher fit participants relative to lower fit participants. Conversely, there were no statistical differences between higher fit and lower fit participants in the right hemisphere. Interestingly, previous studies have reported that exercise increases oscillatory activity in the alpha range during subsequent cognitive performance, often localized to the right frontal hemisphere (Petruzzello and Landers, 1994). This activity is thought to reflect a state of decreased cortical activity associated with relaxation and decreased anxiety (Boutcher, 1993). What the current study adds is the suggestion that levels of physical fitness may also alter brain dynamics in left frontal areas, with lower entropy possibly reflecting higher levels of efficiency in terms of information processing, or the requirements for higher levels of adaptive system uncertainty in the context of executive functioning tasks. Previous studies have shown that task-sensitivity of entropy is associated with poorer cognitive performance when younger and older adults are compared (O’Hora et al., 2013). Specifically, the reduction of EEG entropy from more disordered to less disordered in line with task demands predicted later retrieval. The results of the current study, which focus exclusively on younger cohorts, are consistent with our alternative hypotheses with regards to EEG entropy. Specifically, it may be that greater effort is required by lower fit adolescents, possibly linked to higher levels of information processing complexity and EEG entropy in response to the Erikson flanker task. Alternatively, lower fit adolescents may differ from higher fit adolescents on other biological factors such as their brain metabolism, aspects of fitness that may influence EEG oscillations, attention and vigilance. Further research is needed to explain these EEG entropy differences between higher and lower fitness groups. At the same time, while we also hypothesized that the acute exercise condition would increase cortical processing efficiency and thus result in lower levels of EEG entropy in comparison with the rest condition, we did not find any evidence in support of this hypothesis.

A number of other interesting effects were observed. For example, in the parietal region, results indicated that while entropy reduced significantly from the 0–500 ms post-stimulus interval to the 500–1000 ms interval in both hemispheres, entropy increased significantly from the 500–1000 ms interval to the 1000–1500 ms interval in the right hemisphere, but not in the left hemisphere. Also, in the temporal region, entropy decreased more gradually in the left temporal region than in the right temporal region for both higher fit and lower fit participants. These findings suggest that entropy measures may reveal differential responses of the right and left hemispheres to stimulus processing demands over time and offer a window into hemispheric specialization for different cognitive performance tasks.

As noted above, one interpretation of the frontal lobe EEG entropy findings in the current study is that the lower fit group exerted a greater amount of effort than the higher fit group. The higher levels of cardiorespiratory fitness in the higher fit group may have facilitated greater cortical efficiency, and greater performance efficiency, with fewer cognitive resources needed to maintain performance in comparison with lower fit individuals (Aberg et al., 2009; Stroth et al., 2009; Hillman et al., 2014; Khan and Hillman, 2014). As performance differences between higher fit and lower fit participants were less pronounced after 20 min of aerobic exercise, this suggests that acute exercise might improve cognitive performance efficiency particularly in lower fit individuals (see also Colcombe and Kramer, 2003; McAuley et al., 2013). This interpretation is consistent with the finding that lower fit, but not higher fit adolescents had higher ER for No Go relative to Go trials in the resting condition, whereas in the acute exercise condition there were no differences in ER between groups. This behavioral effect is significant as it suggests that the negative effects of lower levels of physical fitness in adolescents may possibly be ameliorated in the short term by increased aerobic activity, a finding that resonates with the meta-analytical findings of Chang et al. (2012), which found that the cognitive benefits of 20 min of acute exercise are larger for school age children relative to the population as a whole. However, this behavioral effect was not coupled with an effect of acute exercise on EEG entropy in the current study, thus further research is needed to investigate whether or not EEG entropy is sensitive to effects of acute exercise and predictive of changes in cognitive performance that arise in the context of acute exercise manipulations. Although the sample size of the current study was small, which had implications for the power of our statistical analyses, the behavioral and entropy effects observed suggest that lower fit adolescents may perform cognitive tasks at the same level as higher fit participants in certain conditions (exercise condition, go trials), but possibly at the expense of greater cortical effort reflected in higher entropy. Although the task was reasonably difficult in general, with ER of 15–30% across conditions, one possibility is that lower fit participants with higher EEG entropy in left frontal regions may have found different aspects of the task more or less difficult in different conditions. Future research should seek to examine if physical fitness training interventions serve to increase cortical efficiency and cognitive performance, specifically, by altering the entropy of electrical activity across scalp locations.

The effects of aerobic exercise interventions have been examined in the context of both their potential to affect the developmental and scholastic achievement trajectory of children and adolescents, as well as their potential to delay the neurocognitive effects of aging in older adult populations (Erickson et al., 2011; Anderson-Hanley et al., 2012). In this burgeoning field, the effects of physical fitness (Chaddock et al., 2011, 2012b) and exercise interventions have primarily been assessed through behavioral performance measures and the use of neuroimaging modalities such as MRI (Chaddock et al., 2012a; Chaddock-Heyman et al., 2014) and the modulation of ERPs (ERPs) in EEG (Hillman et al., 2011).

Acute bout studies conducted with children have shown that a session of moderate intensity aerobic exercise facilitates resolution of response conflict and processing speed (Drollette et al., 2014), and has selective effects on inhibitory control processes and reading comprehension (Hillman et al., 2009). Acute bout studies with adolescents have reported mixed findings, with some studies indicating that inhibitory processes may no longer be as sensitive to a session of aerobic exercise in adolescence (Stroth et al., 2009), while other studies have reported greater improvements in inhibition and working memory among preadolescents when compared to younger children (Chen et al., 2014). This highlights the need to employ a nuanced developmental approach when considering the effects of exercise and how effects are moderated by age.

Given this perspective, numerous acute bout studies have focused on the cognitive effects and neuroelectric indices of aerobic fitness, in addition to its interaction with aerobic exercise in adolescents. The P3 and N2 are ERP components have been found to be particularly sensitive to the effects of fitness level and exercise. They are associated with the allocation of attentional resources and conflict monitoring, respectively (Patel and Azzam, 2005). For example, Stroth et al. (2009) conducted an acute exercise study with a sample of 35 higher and lower fit adolescents to determine the effects of exercise on response inhibition and task preparation in the same modified version of the Eriksen flanker task used here (Ruchsow et al., 2005). In addition to the N2 and P3 ERP components, the authors were also interested in the contingent negative variation (CNV). This ERP component serves as an index of task preparation, as it reflects anticipation of an upcoming complex cognitive task in response to a stimulus that invokes effortful task preparation in the interval preceding target presentation. The authors observed increased CNV amplitudes in the higher fit adolescents coupled with decreased N2 amplitudes, when compared to their lower fit counterparts. Taken together, their findings indicate aerobic fitness and not an acute bout of exercise facilitates enhanced task preparation and expectancy resulting in more efficient conflict monitoring. Similarly, the current study did not reveal any changes in EEG entropy as a result of acute exercise; rather we found that fitness level influenced cortical processing as evidenced by significantly lower left frontal entropy of the higher fit adolescents when compared to the lower fit adolescents.

Moreover, Pontifex et al. (2011) conducted a study to determine the effects of cardiorespiratory fitness on cognitive control and the flexible adaptation of cognitive resources among preadolescent children completing a modified Flanker task, with incompatible stimulus-response conditions designed to induce even greater interference. The authors were particularly interested in the N2, P3 and ERN components. The ERN, error-related negativity, occurs post error commission and is thought to reflect the activation of action-monitoring processes which recruit further top-down processing in response to an error of commission. They reported enhanced P3 amplitudes, decreases in P3 latency, and a greater modulation of the P3 amplitude between compatible and incompatible conditions in higher fit participants when compared to lower fit participants. Another interesting set of findings indicating more flexible modulation of cognitive control among the higher fit participants relative to lower fit participants included smaller ERN amplitudes in the compatible condition, and greater modulation of the ERN between compatible and incompatible conditions. Again, these findings suggest that the higher fit children were able to more flexibly recruit and adjust cognitive control processes based on task demand and difficultly. One possibility is that lower left frontal entropy for higher fit, relative to lower fit, participants in the current study is related to increased flexibility and control in response to task demands. The idea that physical fitness may relate to EEG entropy points to a possible mechanism underlying fitness-related differences in ERP indices of cognitive flexibility. However, further research is needed to examine these relationships.

As discussed above, there are numerous examples in the extant literature where the effects of aerobic exercise on the P3 component have been investigated. EEG entropy could serve to enrich our understanding of the neural dynamics related to physical activity and its effects on neuroelectric indices related to cognitive functioning. Research conducted by Quiroga et al. (2001) found a significant correlation between decreases in wavelet entropy (WS) and P3 component amplitudes in stimulus-locked intervals of the EEG. The authors contend that this correlation demonstrates the transition of the EEG activity and the multiple frequencies present from a more “disordered state” to a more “ordered state”.

At the same time, there are a number of limitations of the current study that should be noted. First, the sample size in the current study was relatively small and thus effects should be interpreted with caution. The small sample impacted on the power of our analysis and may explain why we observed no significant relationships between entropy levels and indices of cognitive performance. The effects of fitness and acute exercise on brain and behavioral function should be replicated in larger samples of children and adolescents while controlling for a variety of other factors that might be related to both fitness levels and brain and behavioral measures, including socioeconomic status, health status, intelligence, academic achievement, personality and motivation. Also, given that the increased level of arousal induced by physical activity may mediate performance effects observed in cognitive testing situations (Davranche and Audiffren, 2004), future studies need to examine if the arousal effects of exercise differ for children, adolescents and adult samples. Second, future studies should use standardized fitness measures and established norms in efforts to distinguish high fit from low fit groups. More precise measurements such as the oxygen uptake (VO_2_) measure should be used in future studies to categorize fitness groups. While the fitness measurement is a limitation of the current study, we had to analyze fitness levels in the school context and in this context only the continuous graded exercise test performed on a cycle ergometer was possible to estimate student’s fitness levels in relation to BMI.

In conclusion, the present study revealed an interaction between an acute bout of exercise and physical fitness in adolescence. Notably, higher fit, but not lower fit, participants had significantly faster reaction times in the exercise condition in comparison with the rest condition. Furthermore, performance differences between higher fit and lower fit participants were less pronounced after 20 min of aerobic exercise. Specifically, lower fit, but not higher fit, participants showed higher ER for No Go relative to go trials in the rest condition. In the acute exercise condition there were no differences in ER between groups. At the neural level, higher fit participants had lower levels of EEG entropy in left frontal regions, possibly indicating greater efficiency of cognitive resource allocation to the task demands. The results suggest that physical fitness may enhance cognition in adolescence by facilitating higher functionality of the attentional system in the context of lower levels of frontal EEG entropy. The present study also highlights the potential benefits of intervention programs providing physical exercise for adolescents, which may improve attention and cognitive performance at school and in everyday life.

## Conflict of Interest Statement

The authors declare that the research was conducted in the absence of any commercial or financial relationships that could be construed as a potential conflict of interest.
